# Calcitriol restores antiestrogen responsiveness in estrogen receptor negative breast cancer cells: A potential new therapeutic approach

**DOI:** 10.1186/1471-2407-14-230

**Published:** 2014-03-29

**Authors:** Nancy Santos-Martínez, Lorenza Díaz, David Ordaz-Rosado, Janice García-Quiroz, David Barrera, Euclides Avila, Ali Halhali, Heriberto Medina-Franco, María J Ibarra-Sánchez, José Esparza-López, Javier Camacho, Fernando Larrea, Rocío García-Becerra

**Affiliations:** 1 Departments of Reproductive Biology, Instituto Nacional de Ciencias Médicas y Nutrición Salvador Zubirán, Vasco de Quiroga No. 15, Tlalpan 14000 México, México; 2 Department of Pharmacology, Centro de Investigación y de Estudios Avanzados, I.P.N., Av. Instituto Politécnico Nacional 2508 Gustavo A. Madero, 07360 México, D.F, México; 3 Department of Surgery, Instituto Nacional de Ciencias Médicas y Nutrición Salvador Zubirán, Vasco de Quiroga No. 15, Tlalpan 14000 México, D.F, México; 4 Biochemistry Unit. Instituto Nacional de Ciencias Médicas y Nutrición Salvador Zubirán, Vasco de Quiroga No. 15, Tlalpan 14000 México, D.F, México

**Keywords:** Estrogen receptor, Breast cancer, Hormonal therapy, Calcitriol, VDR

## Abstract

**Background:**

Approximately 30% of breast tumors do not express the estrogen receptor (ER) α, which is necessary for endocrine therapy approaches. Studies are ongoing in order to restore ERα expression in ERα-negative breast cancer. The aim of the present study was to determine if calcitriol induces ERα expression in ER-negative breast cancer cells, thus restoring antiestrogen responses.

**Methods:**

Cultured cells derived from ERα-negative breast tumors and an ERα-negative breast cancer cell line (SUM-229PE) were treated with calcitriol and *ERα* expression was assessed by real time PCR and western blots. The ERα functionality was evaluated by prolactin gene expression analysis. In addition, the effects of antiestrogens were assessed by growth assay using the XTT method. Gene expression of cyclin D1 (*CCND1*), and Ether-à-go-go 1 (*EAG1*) was also evaluated in cells treated with calcitriol alone or in combination with estradiol or ICI-182,780. Statistical analyses were determined by one-way ANOVA.

**Results:**

Calcitriol was able to induce the expression of a functional ERα in ER-negative breast cancer cells. This effect was mediated through the vitamin D receptor (VDR), since it was abrogated by a VDR antagonist. Interestingly, the calcitriol-induced ERα restored the response to antiestrogens by inhibiting cell proliferation. In addition, calcitriol-treated cells in the presence of ICI-182,780 resulted in a significant reduction of two important cell proliferation regulators *CCND1* and *EAG1*.

**Conclusions:**

Calcitriol induced the expression of ERα and restored the response to antiestrogens in ERα-negative breast cancer cells. The combined treatment with calcitriol and antiestrogens could represent a new therapeutic strategy in ERα-negative breast cancer patients.

## Background

Breast cancer is a heterogeneous disease, encompassing a number of distinct biological entities that are associated with a variety of pathological and clinical features [1]. The gene expression profile of breast cancer allows to classify this disease in five groups, two of them estrogen receptor (ER)-positive (luminal A and B) and three ER-negative (normal breast-like, human epidermal growth factor receptor- 2 (HER2) and basal-like) [2]. Approximately 30% of all breast tumors do not express ER, a protein with both prognostic and predictive values. Indeed, the presence of ERα correlates with increased disease-free survival and better prognosis. Importantly, ERα-positive breast cancers respond appropriately to endocrine therapies [3-5]. Tamoxifen is the most common and effective therapy in pre- and postmenopausal patients affected with ER-positive tumors, since a long-term use of this compound increases disease-free survival and reduces tumor recurrence [6,7]. Unfortunately, up to 50% of patients bearing ERα-positive primary tumors lose receptor expression in recurrent tumors, and about one third of metastatic tumors develop resistance to tamoxifen and lose ERα expression [8]. The lack of ER expression has been linked to epigenetic mechanisms or to others such as hyperactivation of the mitogen-activated protein kinase (MAPK) signaling pathway or increased expression of specific microRNAs [9-11]. In fact, knockdown of specific microRNAs or inhibition of MAPK activity is followed by restoration of a functional ERα in ER-negative breast cancer cells [9,10]. These findings indicate that the ERα-negative phenotype could be reverted for therapeutic purposes.

Calcitriol, the most active metabolite of vitamin D, elicits significant antiproliferative activity in breast cancer cells by several vitamin D receptor (VDR) mediated mechanisms including regulation of growth arrest, cell differentiation, migration, invasion and apoptosis [12-14]. Epidemiological studies have demonstrated an association between low levels of calcidiol, the precursor of calcitriol, and increased risk of developing breast cancer [15]. Moreover, low levels of calcitriol are associated with disease progression and high incidence of ER-negative and triple-negative breast tumors [16,17], while VDR-positive breast cancer patients had significantly longer disease-free survival than those with VDR-negative tumors [18]. Indeed, VDR knock-out mice are more likely to develop ER- and progesterone receptor (PR)- negative mammary tumors as compared with their wild type littermates [17], highlighting calcitriol prodifferentiating properties. Our laboratory and other groups have demonstrated the potent antipropiferative activity of calcitriol in cells derived from biopsies or in established cell lines from breast cancer [19-21]. Additionally, other studies have demonstrated the antiproliferative effects of vitamin D compounds in ER-responsive human breast cancer cells through downregulation of ER and disruption of estrogen dependent signaling pathways [20,22,23]. However, calcitriol also inhibited proliferation in ER-negative cell lines, suggesting that growth inhibition induced by calcitriol is not solely mediated through the ER [12]. In this regard, ERα regulation studies in several human breast cancer cell lines showed that calcitriol treatment decreased or did not modify ER expression [20,22-24]. In contrast, in an ER-negative breast cancer cell line calcitriol increased estrogen binding proteins [24].

In order to increase our knowledge concerning the participation of calcitriol in ER regulation, the aim of the present study was to investigate if this hormone induces a functional ER and consequently could restore the antiproliferative effects of antiestrogens in ER-negative breast cancer cells.

## Methods

### Reagents

Estradiol (E_2_), 4-hydroxytamoxifen and calcipotriol (MC 903) were purchased from Sigma (St. Louis, MO, USA). Cell culture medium was obtained from Life Technologies (Grand Island, NY, USA). Fetal bovine serum (FBS) was from Hyclone Laboratories Inc. (Logan, UT, USA) and the antiestrogen ICI-182,780 (Fulvestrant) from Zeneca Pharmaceuticals (Wilmington, DE, USA). Gefitinib (Iressa, ZD1839) was kindly provided by AstraZeneca (Wilmington, DE, USA). U0126 was from Millipore (MA, USA). Trizol and the oligonucleotides for real time polymerase chain reaction (qPCR) were from Invitrogen (CA, USA). The TaqMan Master reaction, probes, capillaries, reverse transcription (RT) system and the cell proliferation assay (XTT) were purchased from Roche (Roche Applied Science, IN, USA). MCF-7 nuclear extract was purchased from Santa Cruz Biotechnology Inc., (CA, USA). The VDR antagonist (23S)-25-dehydro-1-hydroxyvitamin D3-26,23-lactone (TEI-9647) and 1α,25-dihydroxycholecalciferol (calcitriol) were kindly donated from Teijin Pharma Limited (Tokyo, Japan) and Hoffmann-La Roche Ltd. (Basel, Switzerland), respectively.

### Human tissues

The protocol was approved by the Institutional Review Board “Comité Institucional de Investigación Biomédica en Humanos (No. 1967, 2009)” of the “Instituto Nacional de Ciencias Médicas y Nutrición Salvador Zubirán (Mexico City). Before mammary biopsies donation, all participating patients signed an informed consent. Biopsies were obtained from patients with ER-negative breast cancer. The samples were harvested and processed as described previously [19]. A total of 5 independent cultured specimens were used for this study. The ER-negative SUM-229PE (Asterand, San Francisco, CA) and the ER-positive BT-474 (ATCC) and MCF-7 (ATCC) established cell lines were also studied.

### Cell culture

Primary tumor cultures were derived from biopsies of breast cancer patients as described previously [19,25]. The cells were cultured in DMEM-HG medium supplemented with 5% heat-inactivated-FBS, 100 U/ml penicillin, 100 μg/ml streptomycin; and incubated in 5% CO_2_ at 37°C. After approximately 8 passages cells were characterized by western blot and immunocytochemistry. Established cell lines were maintained according to indications from suppliers. All experimental procedures were performed in DMEM-F12 medium supplemented with 5% charcoal-stripped-heat-inactivated FBS, 100 U/ml penicillin and 100 μg/ml streptomycin.

### Immunocytochemistry

Cultured cells were grown on glass coverslips and fixed in 96% ethanol. Antigen retrieval was done by autoclaving in EDTA decloaker 5× solution (pH 8.4-8.7, Biocare Medical, CA, USA) during 10 min. Slides were blocked with immunodetector peroxidase blocker (Bio SB, CA, USA) and incubated with ERα (1:250, Bio SB) [26] and VDR antibodies (1:100, Santa Cruz Biotechnology Inc, CA, USA) [27]. After washing, the slides were sequentially incubated with immune-Detector Biotin-Link and Immuno-Detector HRP label (Bio SB) during 10 min each. Staining was completed with DAB and 0.04% H_2_O_2_.

### Western blots

Cells were incubated in the presence of calcitriol (1X10^-8^ M and 1X10^-7^ M), MAPK inhibitors (U0126; 10 μM, Gefitinib; 0.8 μM) or the vehicle alone during 72 hr. Afterwards, whole-cell protein lysates were prepared using lysis buffer (50 mM Tris-HCl, 150 mM NaCl, 1% Nonidet P-40, pH 7.5) in the presence of a protease inhibitor cocktail. Protein concentrations were determined using the Protein Assay Dye Reagent Concentrate (Bio-Rad, Hercules, CA, USA). The proteins were separated on 10% SDS-PAGE and transferred to nitrocellulose membranes. The membranes were blocked with 5% skim milk and incubated overnight at 4°C in the presence of mouse anti-ERα (1:200, Santa Cruz) [28]. The membranes were washed and incubated with goat anti-mouse HRP-conjugated secondary antibody (1:2000, Santa Cruz). For visualization, membranes were processed with BM chemiluminescence blotting substrate (Roche Applied Science, IN, USA). For normalization, blots were stripped in boiling stripping buffer (2% w/v SDS, 62.5 mM Tris-HCl pH 6.8, 100 mM 2- mercapto-ethanol) for 30 min at 50°C and sequentially incubated with mouse anti-GAPDH (1:10000, Millipore) [29] and anti-mouse-HRP (1:10000, Jackson ImmunoResearch Laboratories, Inc., West Grove, PA, USA). Densitometric analysis of resulting bands was performed by using ImageJ software (NIH, USA).

### Cell proliferation assay

The cells were seeded in 96-well tissue culture plates at a density of 500-1000 cells/well by sextuplicate. After incubating for 24 hr, cells were incubated in the presence or absence of calcitriol (1X10^-8^ M) during 48 hr. Afterwards, culture medium was removed and incubations with E_2_ (1X10^-8^ M), as an ER agonist, or tamoxifen (1X10^-6^ M) and ICI-182,780 (1X10^-6^ M), as ER antagonists, or their combination were performed in the absence or presence of calcitriol. Plates were incubated at 37°C for 6 days and cell viability was determined by using the colorimetric XTT Assay Kit (Roche) according to manufacturer’s instructions. After 4 hr incubation, absorbance at 492 nm was measured in a microplate reader (BioTek, Winooski, VT, USA).

### Real time RT-PCR

For *ERα* gene expression analysis the cells were incubated in the presence of different calcitriol concentrations or the vehicle alone (0.1% ethanol) during 24 hr. In order to establish the participation of the VDR on calcitriol effects upon the *ERα*, the VDR antagonist TEI-9647 (1X10^-6^ M) was coincubated with calcitriol in some experiments. Gene expression analyses of prolactin (*PRL*), cyclin D1 (*CCND1*) and the potassium channel Ether-à-go-go (*EAG1*) were also performed. For this, the cells were treated with calcitriol (1X10^-8^ M) during 48 hr. Afterwards, E_2_ (1X10^-8^ M) or ICI-182,780 (1X10^-6^ M) were added to the culture media and the incubations proceeded for additional 24 hr. Next, RNA was extracted with Trizol reagent and then subjected to reverse transcription using the transcriptor RT system. Real-time PCR was carried out using the LightCycler 2.0 from Roche (Roche Diagnostics, Mannheim, Germany), according to the following protocol: activation of Taq DNA polymerase and DNA denaturation at 95°C for 10 min, proceeded by 45 amplification cycles consisting of 10 s at 95°C, 30 s at 60°C, and 1 s at 72°C. The following oligonucleotides were used: *ERα*-F, CCTTCTTCAAGAGAAGTATTCAAGG; *ERα*-R, GTTTTTATCAATGGTGCACTGG; *EAG1*-F, CCTGGAGGTGATCCAAGATG; *EAG1*-R, CCAAACACGTCTCCTTTTCC; *CCND1*-F, GAAGATCGTCGCCACCTG; *CCND1*-R, GACCTCCTCCTCGCACTTCT; *PRL*-F, AAAGGATCGCCATGGAAAG; *PRL*-R, GCACAGGAGCAGGTTTGAC. The gene expression of the housekeeping gene glyceraldehyde-3-phosphate dehydrogenase (*GAPDH*) *GAPDH*-F, AGCCACATCGCTGAGACAC; *GAPDH*-R, GCCCAATACGACCAAATCC was used as an internal control. Stimulatory concentration (EC_50_) values were obtained by non-linear regression analysis using sigmoidal fitting with a dose-response curve by means of a scientific graphing software (SigmaStat, Jandel Scientific).

### Statistical analyses

Data are expressed as the mean ± standard deviation (S.D.). Statistical analyses were determined by one-way ANOVA followed by the Holm-Sidak method, using a specialized software package (SigmaStat, Jandel Scientific). Differences were considered significant at *P* ≤ 0.05.

## Results

### Calcitriol induced *ERα* expression through a VDR-dependent mechanism in ER-negative breast cancer cells

**Figure 1:**
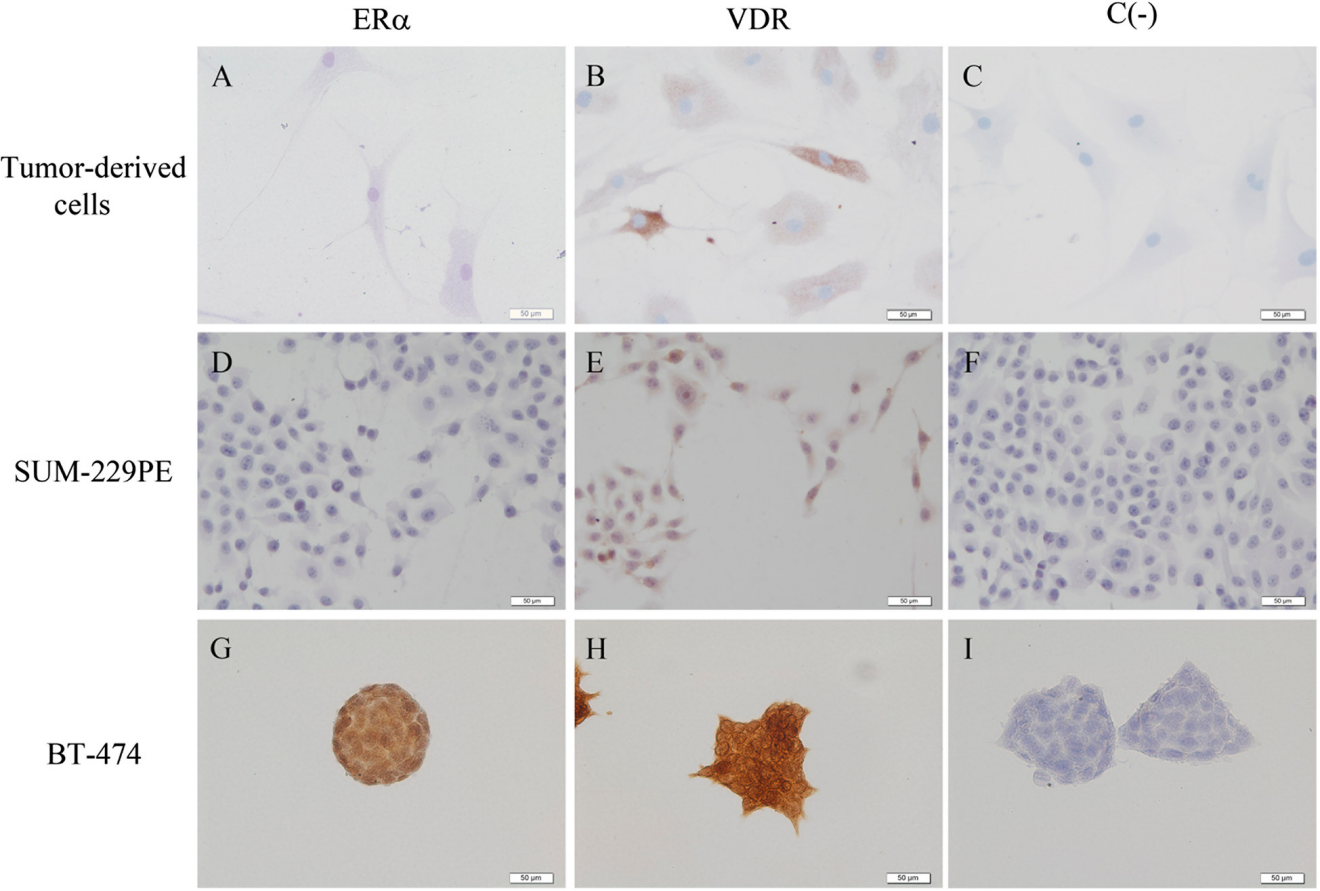
**Immunocytochemical analysis of ERα and VDR in primary and established breast cancer cells.** Representative images of cultured tumor-derived **(A**-**C)**, SUM-229PE **(D-F)** and BT-474 **(G-I)** cells are shown. Tumor-derived **(A)** and SUM-229PE **(D)** cells were negative for ERα, while BT-474 was ERα positive **(G)**. All cells were positive for VDR **(B**, **E** and **H)** in the cytoplasmic, nuclear and perinuclear regions (brown staining). Negative controls were carried out in the absence of primary antibody for each cell line **(C**, **F** and **I)**. Representative pictures are displayed (20 ×).

**Figure 2:**
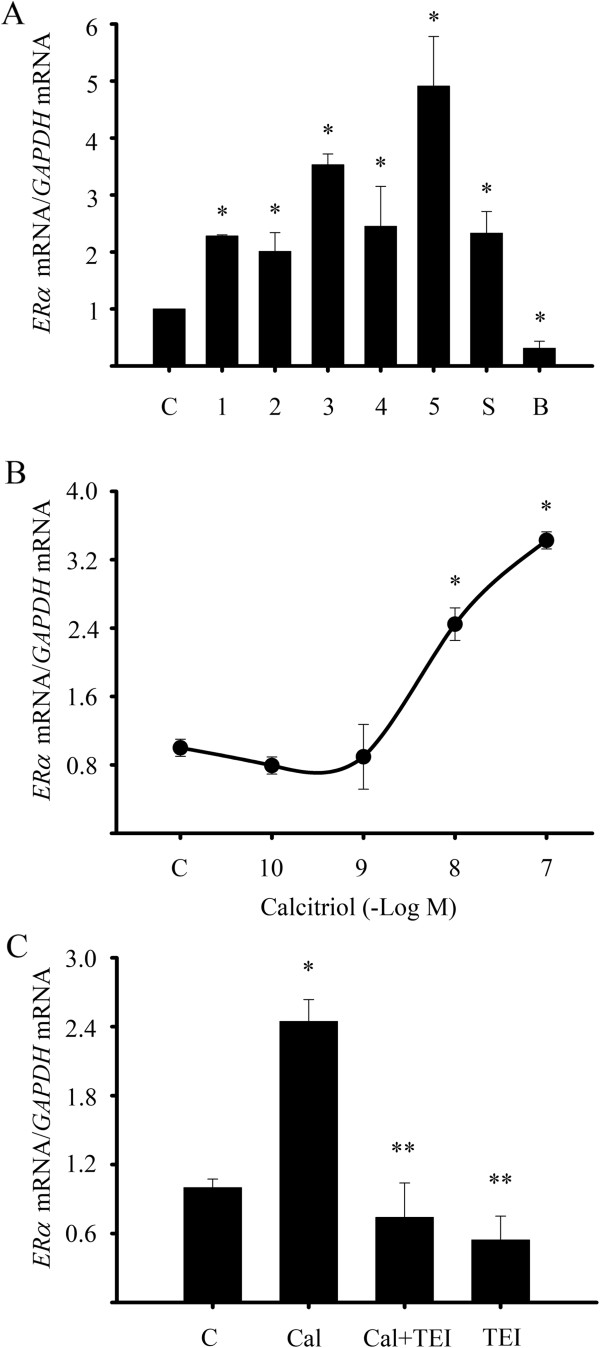
**Calcitriol induced *ERα *mRNA expression through the VDR in ER**α-**negative breast cancer cells. A)** Cultured tumor-derived cells from five patients (1-5) with ERα-negative breast cancer and the ER-negative SUM-229PE (S) and ER-positive BT-474 (B) established cell lines were incubated with calcitriol (1X10^-7^ M) or its vehicle (C, ethanol) for 24 hr. Subsequently, mRNA was extracted and real time RT-PCR (qPCR) was performed. **B)** Cultured breast tumor-derived cells were treated with increasing calcitriol concentrations (1X10^-10^ M and 1X10^-7^ M) for 24 hr. **C)** Cells were incubated in the absence (C) or presence of calcitriol (Cal, 1X10^-8^ M), without or with a VDR antagonist (TEI, 1X10^-6^ M). Results shown are the mean ± S.D. of *ERα*/*GAPDH* mRNA normalized ratio of two independent experiments per triplicate. Data were normalized to 1 for vehicle-treated cells. *P ≤ 0.05 *vs*. C. **P ≤ 0.05 *vs*. calcitriol alone.

Biopsies from five patients with ER-negative breast cancer were obtained and used for cell culturing. These biopsies had a diagnosis of invasive ductal carcinoma and ranged between 5 and 9 in the Scarff-Bloom-Richardson system score. All cultured breast tumor-derived cells were positive for VDR and further confirmed to be negative for ERα (Figure 1). In addition, the ER-negative SUM-229PE and ER-positive BT-474 established cell lines were also studied. All cell lines were incubated in the presence of calcitriol (1X10^-7^ M) during 24 hr and *ERα* gene expression was assessed by qPCR. As shown in Figure 2A, calcitriol significantly induced *ERα* mRNA expression in all tumor-derived cultured cells and SUM-229PE cells. In contrast, calcitriol downregulated *ERα* mRNA levels in BT-474 as it has been previously reported [30].

As shown in Figure 2B, calcitriol significantly increased *ERα* mRNA in a dose dependent manner with an EC_50_ of 9.8X10^-9^ M. This effect was specifically mediated through the VDR, since the VDR antagonist TEI-9647 significantly abolished the stimulatory effect of calcitriol upon *ERα* gene expression. The presence of the VDR antagonist by itself did not modify *ERα* gene expression (Figure 2C).

**Figure 3:**
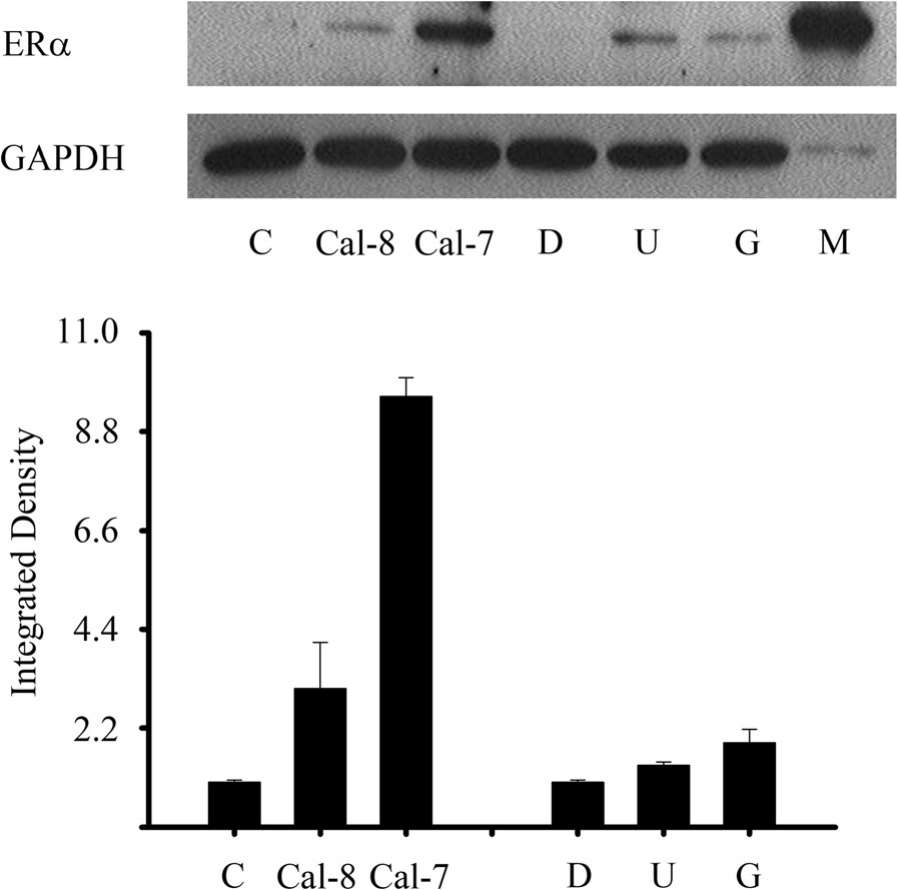
**Calcitriol induced ERα protein expression.** SUM-229PE cells were treated with two calcitriol concentrations (Cal, 1X10^-8^ M and 1X10^-7^ M) and two MAPK inhibitors: U0126 (U, 10 μM) or Gefitinib (G, 0.8 μM) used as controls of ERα induction during 72 hr. Control incubations were done in the presence ethanol (C) or DMSO (D). Results were analyzed by western blots. MCF-7 (M) nuclear extracts were used as positive control for ERα and GAPDH was utilized as the loading control for normalization. Results are representative from two independent experiments.

In order to assess if calcitriol induced ERα protein expression, the SUM-229PE cell line was incubated in the presence of calcitriol and western blot analyses were performed. Figure 3 shows the results of cells incubated with two calcitriol concentrations (1X10^-8^ and 1X10^-7^ M) during 72 hr. The presence of a 66 KDa band corresponding to ERα, as judged by the positive control in MCF-7 cells, was observed in calcitriol-treated cells. Moreover, a higher calcitriol concentration further increased the relative abundance of ERα as shown in Figure 3. Inhibitors of the MAPK signaling pathway (U0126 and Gefitinib) were used as controls of ERα induction [10].

### Calcitriol induced a functional ERα

**Figure 4:**
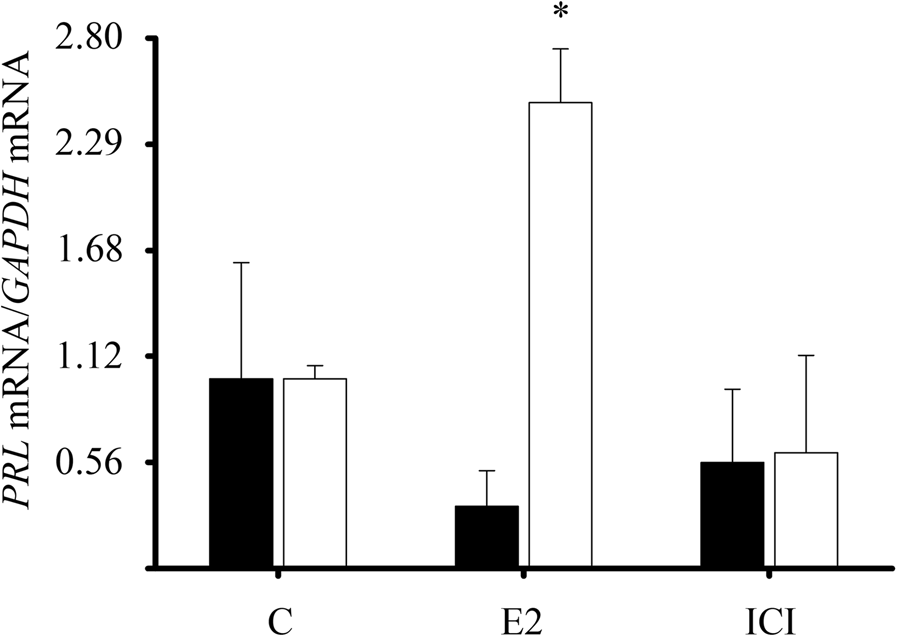
**Calcitriol induced a fully active ERα.** Cultured breast tumor-derived cells were incubated in the absence (black bars) or presence of calcitriol 1X10^-8^ M (white bars) for 48 h. Subsequently, cells were coincubated with or without calcitriol plus estradiol (E_2_, 1x10^-8^ M), ICI-182,780 (ICI, 1x10^-6^ M) or vehicle (C) for 24 h. *PRL* gene expression was determined by qPCR. Results are shown as the mean ± S.D. of *PRL*/*GAPDH* mRNA normalized ratio. Data were normalized to 1 for vehicle-treated cells. *P ≤ 0.05 *vs*. C.

In order to determine the functionality of the ERα induced by calcitriol, we evaluated the effects of E_2_ and the antiestrogen ICI-182,780 on the expression of *PRL*, cathepsin D (*CTSD*) and trefoil factor 1 (*TFF1*) as examples of estrogen inducible genes [31]. Breast tumor-derived cells were cultured first in the presence or absence of calcitriol (1X10^-8^ M) during 48 hr and subsequently incubated in the presence of E_2_ (1X10^-8^ M) or ICI-182,780 (1X10^-6^ M) with or without calcitriol for 24 hr (Figure 4). In the absence of calcitriol (black bars), E_2_ and ICI-182,780 did not modify *PRL* mRNA; however, in calcitriol-treated cells (white bars), E_2_ significantly upregulated *PRL* expression. The presence of the antiestrogen alone did not change *PRL* gene expression. These data suggest that the calcitriol-induced ERα is a fully-transcriptionally active receptor. Interestingly, calcitriol *per se* significantly stimulated the expression of both *CTSD* and *TFF1* genes, which may explain why E2 was not able to further increase gene expression (data not shown).

### Calcitriol restored the antiestrogenic response in ERα-negative breast cancer cells

**Figure 5:**
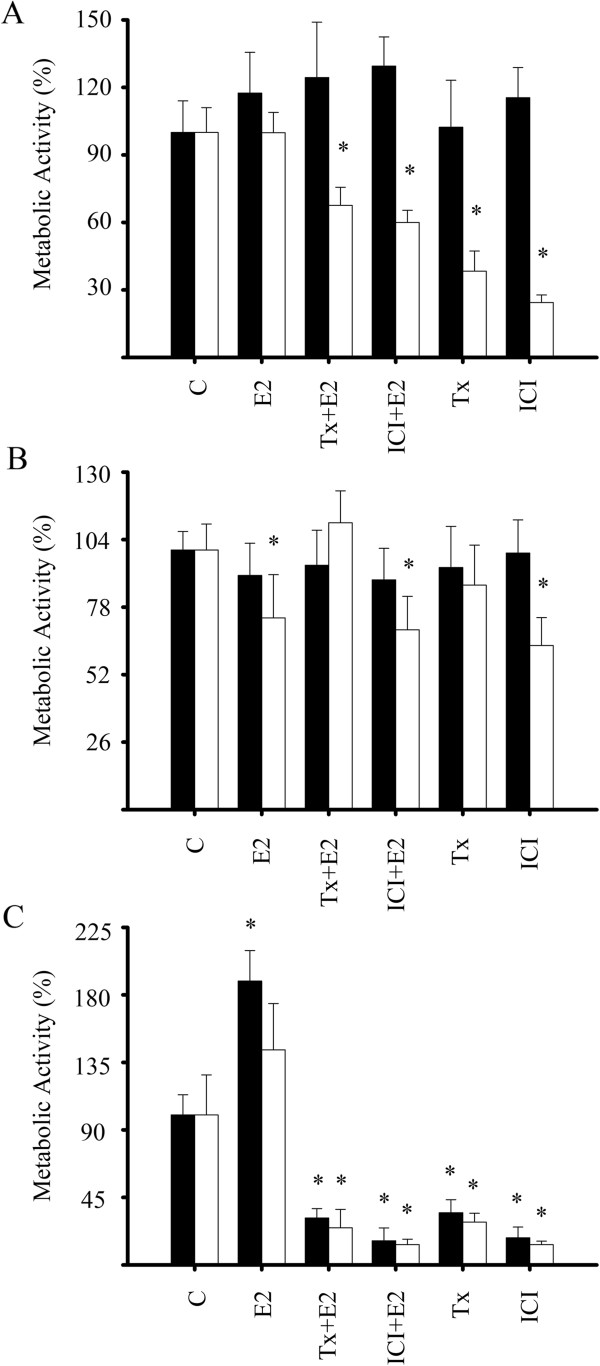
**ER**α **induction restored the response to antiestrogens in ER**-**negative breast cancer cells. A)** Cultured breast tumor-derived cells, **B)** SUM-229PE and **C)** MCF-7 were incubated in the absence (black bars) or presence of calcitriol 1X10^-8^ M (white bars) for 48 h. Afterwards, cells were coincubated without (black bars) or with calcitriol (white bars) plus estradiol (E_2_, 1X10^-8^ M), tamoxifen (Tx, 1X10^-6^ M), ICI-182,780 (ICI, 1X10^-6^ M), ethanol (C), or combination of antagonists with E_2_ for 6 days. Cell growth assays by the XTT colorimetric method were performed. Bars represent the mean ± S.D. Data were normalized to 100% using the activity of vehicle-treated cells. Results are representative from two independent experiments performed in sextuplicates.* *P* ≤ 0.05 *vs*. control for each group (black bars *vs* black control or white bars *vs* white control).

In order to assess whether the calcitriol-induced ERα was sensitive to the antiproliferative effects of the antiestrogens in ERα-negative breast cancer cells, growth assays were performed. Breast cancer cells were incubated in the presence of calcitriol (1X10^-8^ M) or the vehicle alone for 48 hr. Afterwards, cells were incubated with ER agonist (1X10^-8^ M), antagonists (1X10^-6^ M) or the combination of E_2_ plus antagonists during 6 days. The results demonstrated that in the absence of calcitriol (black bars), none of the compounds affected cell growth in both cultured breast tumor-derived cells (Figure 5A) and the SUM-229PE cell line (Figure 5B). Interestingly, in calcitriol-treated tumor-derived cells (white bars), antiestrogens alone or in combination with E_2_ significantly inhibited cell proliferation as compared with control cells (C, white bar). The presence of E_2_ at the dose of 1X10^-8^ M did not modify cell growth (Figure 5A); however, higher E_2_ concentrations (1X10^-7^ M) significantly inhibited cell growth (data not shown). Similar results were observed in SUM-229PE cells, but tamoxifen alone or in combination with E_2_ did not affect cell growth (Figure 5B). MCF-7 cells were used as control of the inhibitory effect of the antiestrogens *via* ERα (Figure 5C). As depicted, E_2_ significantly increased cell proliferation in cells not treated with calcitriol; however, this effect was not observed in those cells cultured in the presence of calcitriol, most likely due to its antiproliferative activity. As expected, antiestrogens and their combination with E_2_ significantly inhibited cell growth in both treated and not-treated calcitriol cells.

### Antiestrogen treatment downregulated *CCND1* and *EAG1* gene expression in calcitriol-treated breast cancer cells

**Figure 6:**
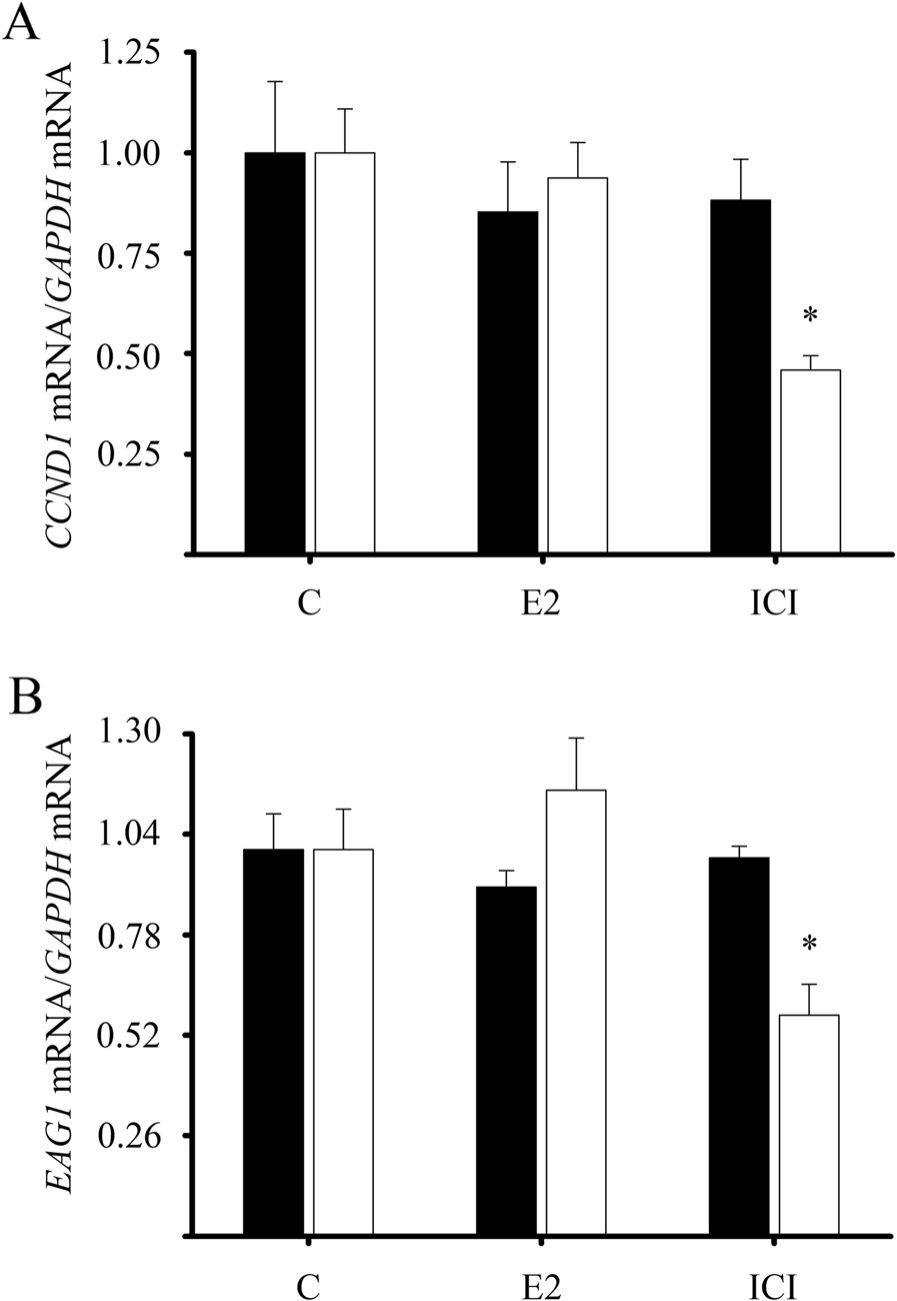
**ICI**-**182**,**780 downregulated *CCND1 *and *EAG1 *gene expression in calcitriol**-**treated ERα-negative cells.** Cultured breast tumor-derived cells were incubated in the absence (black bars) or presence of calcitriol 1X10^-8^ M (white bars) for 48 h. Subsequently, cells were coincubated with or without calcitriol plus estradiol (E_2_, 1x10^-8^ M), ICI-182,780 (ICI, 1X10^-6^ M) or its vehicle (C) for 24 hr. **A)***CCND1* and **B)***EAG1* gene expression was determined by qPCR. Results shown are the mean ± S.D. of *CCND1* or *EAG1*/*GAPDH* mRNA normalized ratio. Data were normalized setting a value of 1 for vehicle-treated cells. *P ≤ 0.05 *vs*. C.

One of the molecular mechanisms by which antiestrogens inhibit cell proliferation is by decreasing *CCND1* expression and blockage of cell cycle progression *via* the ER [32,33]. Thus, we studied the effects of ICI-182,780 and E_2_ on *CCND1* expression in calcitriol-treated ERα-negative breast tumor-derived cells. As shown in Figure 6A, only in calcitriol-treated cells the presence of ICI-182,780 (1X10^-6^ M) but not E2 downregulated *CCND1* gene expression.

In breast cancer cell lines the inhibition of *EAG1* potassium channel expression is accompanied by a significant reduction of cell proliferation [19,34]. Therefore, we evaluated the effects of an agonist or antagonist of the calcitriol-induced ER on *EAG1* expression. As shown in Figure 6B, neither E_2_ nor ICI-182,780 altered *EAG1* gene expression in non-calcitriol treated cells (black bars); however, when compared with cells in the presence of calcitriol, the antiestrogen, in contrast to E2 alone, significantly decreased *EAG1* mRNA levels (white bars).

### Calcipotriol, a vitamin D analogue, increased *ERα* expression

**Figure 7:**
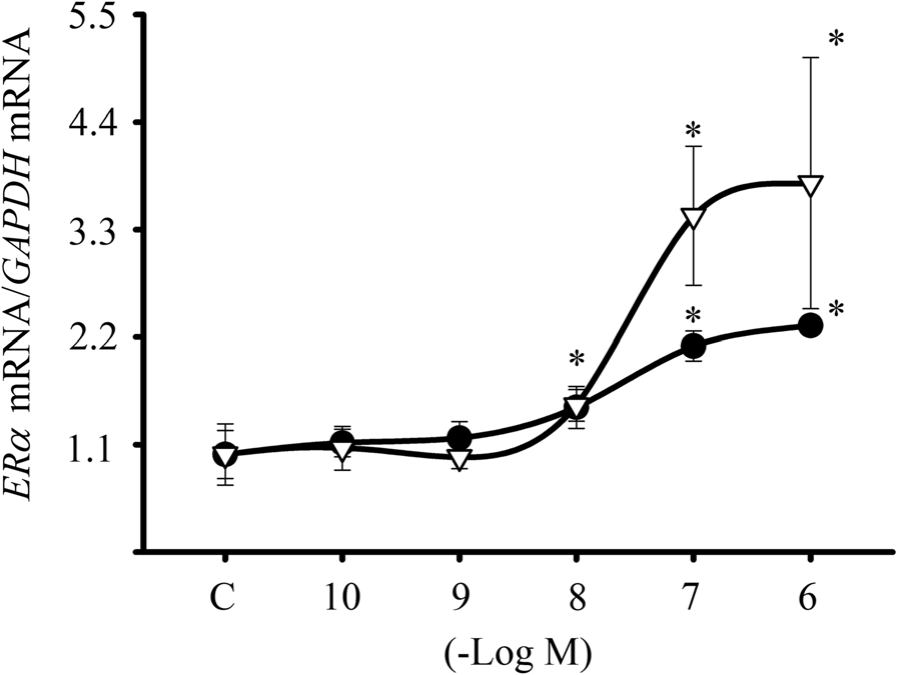
**Calcipotriol induced *ERα *expression in ER**-**negative breast cancer cells.** SUM-229PE cells were cultured in the presence of different calcitriol (circles) and calcipotriol (triangles) concentrations or vehicle alone (C, ethanol) for 24 hr. Afterwards, mRNA was extracted and qPCR was performed. Results are shown as the mean ± S.D. of *ERα*/*GAPDH* mRNA normalized ratio. Data were normalized setting a value of 1 for vehicle-treated cells. *P ≤ 0.05 *vs.* C.

Calcipotriol, a synthetic low calcemic vitamin D analogue, has been considered a potent stimulator of cell differentiation and inhibitor of cell proliferation in cancer cells [35]. Figure 7 shows a comparison between different concentrations of calcipotriol and calcitriol (1X10^-10^ to 1X10^-6^ M) upon *ERα* gene expression in SUM-229PE. As depicted, both compounds increased *ERα* gene expression in a concentration-dependent manner with similar EC_50_ values (2.74X10^-8^ M and 2.21X10^-8^ M, for calcipotriol and calcitriol, respectively).

## Discussion

In breast cancer, the presence of the ERα is considered as a good indicator of disease-free survival and prognosis since patients with ERα-positive tumors are candidates for hormonal therapy [3,4,6]. In contrast, tumors lacking this receptor have the poorest clinical prognosis [36]. In this study we demonstrated the ability of calcitriol to induce the expression of ERα in both primary and established ERα-negative breast cancer cell lines. This effect was mediated by a VDR-dependent mechanism. In addition, our results demonstrated a fully active calcitriol-induced ER by its ability to increase *PRL gene* expression. Interestingly, pretreatment of ER-negative breast tumor-derived cells with calcitriol and the further incubation with this secosteroid in combination with tamoxifen or ICI-182,780 resulted in a significantly lower cell growth proliferation.

It is noteworthy to mention that, to our knowledge, this study is the first to demonstrate the ability of calcitriol to induce the expression of a functional ERα in both primary and established ERα-negative breast cancer cells, which we think is of biological importance given its potential for future treatment strategies to improve prognosis in ERα-negative breast cancer patients.

Since it has been observed that MAPK inhibitors increase ERα protein in ER-negative breast tumor cells [10], we hypothesized that the upregulation of ERα by calcitriol could be the result of decreased MAPK activity. Although, in this study we could not demonstrate any change in this kinase in the presence of calcitriol. An alternative, mechanism by which calcitriol *via* its receptor induced ERα expression might be at the level of promoter-driven transcriptional regulation. Therefore, in order to identify putative vitamin D response elements we performed an *in silico* analysis with the MatInspector software [37] using a sequence derived from the human chromosome 6, which contains the promoter region of ERα [38]. The results from this analysis showed the presence of several putative vitamin D response elements of the DR3 and DR4 types, supporting the idea of a direct transcriptional regulation of ER promoter by calcitriol.

The observation that tamoxifen and ICI-182,780 inhibited cell growth in calcitriol-treated ER-negative breast tumor-derived cells indicated the induction of a functionally active ERα. However, cell growth inhibition by tamoxifen was not observed in the case of calcitriol-treated ER-negative SUM-229PE cells. This finding might be explained as a receptor resistance–like condition resulting probably from the hyperactivation of the MAPK signaling pathway due to overexpression of EGFR or HER2 as has been previously observed in breast cancer cells [10].

It is well known that E_2_ exhibits proliferative effects and therefore stimulates tumor growth in breast cancer [39,40]. However, in the present study, the presence of E_2_ did not result in increased proliferation of cells pretreated with calcitriol. It is possible that the lack of mitogenic activity of E_2_ through the newly expressed ERα was due to a priming antiproliferative effect of calcitriol, thus preventing the expected estradiol-mediated effects on cell proliferation. This observation agreed with those of Bayliss *et al*., [10] who showed that E2 did not increase proliferation in cells where the ERα was reexpressed by MAPK inhibitors, including in those studies in ER-negative breast cancer cells transfected with the ER [41].

In this study, the ability of antiestrogens to inhibit cell growth in an estradiol-depleted condition might require further investigation; however, some effects of these compounds on the mitogenic activity of growth factors, in the absence of estrogens have been already demonstrated in breast cancer [33,42]. In this regard, one of the most common regulators known to be altered and overexpressed in various cancers including breast is CCND1, which functions as mitogenic sensor and allosteric activator of cyclin-dependent kinase (CDK)4/6 [43]. It is known that the inhibitory actions of antiestrogens on breast cancer are in part exerted through the downregulation of *CCND1*[33]. In this study, the results showing that ICI-182,780 significantly decreased *CCND1* mRNA only in calcitriol-treated cells, indicated that these compounds may affect cell cycle regulation as has already been shown in ER-positive breast tumors [33]. Furthermore, the demonstration of a significant inhibition of *EAG1* gene expression by ICI-182,780 in calcitriol-treated cells, suggested that the antiproliferative effects of these compounds involve a number of regulatory mechanisms which are under the control of ERα activation. These results suggest that calcitriol in combination with ICI-182,780, through downregulation of *EAG1* and *CCND1* affect cell proliferation and tumor progression [34,44].

There are several markers associated with tumor aggressiveness. Among these, myoepithelial markers, which are preferentially expressed in ER-negative breast cancer, suggest that the loss of the steroid receptor is related to the degree of cellular dedifferentiation occurring in these tumors [45]. It is known that calcitriol promotes differentiation of several tumor cell types, including human breast and colon cancers [14,46]. This process involves the action of calcitriol on a number of events, such as the induction of adhesion proteins (E-cadherin, claudin, occludin) or by interfering with some intracellular signaling pathways, such as the Wnt/b-catenin signaling [14,46]. Our results revealed that calcitriol induced ERα gene and protein expression suggesting that calcitriol affects the phenotype of ERα-negative breast cancer cells by reverting cellular mechanisms associated with a more aggressive behavior and poor prognosis.

The development of numerous vitamin D analogues and intermittent calcitriol dosing have allowed substantial dose-escalation and reduced calcemic effects [47,48]. Calcipotriol, a synthetic vitamin D analogue with a significantly lower calcemic effect, is also known as a potent antiproliferative compound and an inducer of cell differentiation [35]. In this study, the demonstration that calcipotriol was also able to upregulate *ERα* gene expression in an ER-negative breast cancer cell line, suggest that treatment options in breast cancer patients might also include vitamin D analogues with reduced side calcemic effects.

Our results suggest that the use of calcitriol in combination with aromatase inhibitors or ER antagonists might be considered in the future as a new strategy for the treatment of ERα-negative breast cancer, including the triple-negative subtypes.

## Conclusions

The results presented herein clearly demonstrated the ability of calcitriol and its synthetic analog calcipotriol to upregulate ERα expression in a subset of ER-negative breast cancer cells. These results may offer a therapeutic alternative, particularly in those patients affected with ER-negative tumors by sensitizing them to hormone therapy, with the aim at improving disease prognosis.

## Abbreviations

CTSD: Cathepsin D; CCND1: Cyclin D1; E2: Estradiol; EAG1: Ether-à-go-go 1; EC50: Stimulatory concentration; EGFR: Human epidermal growth factor receptor- 1; ER: Estrogen receptor; FBS: Fetal bovine serum; GAPDH: Glyceraldehyde-3-phosphate dehydrogenase; HER2: Human epidermal growth factor receptor- 2; MAPK: Mitogen-activated protein kinase; PR: Progesterone receptor; PRL: Prolactin; qPCR: Real time polymerase chain reaction; RT: Reverse transcription; S.D: Standard deviation; TFF1: Trefoil factor 1; VDR: Vitamin D receptor.

## Competing interests

The authors declare that they have no competing interests.

## Authors’ contributions

RGB and LD were involved in the conception, design and coordination of the study as well as in data analysis, interpretation of results, actively participated in all experimental procedures, and were involved in drafting the manuscript. NSM was in charge of all experimental procedures, participated in data analysis and interpretation, as well as in drafting the manuscript. DOR, JGQ, DB, MJIS and JEL participated in the experimental procedures and revised critically the content of the manuscript. HMF provided breast biopsies, carried out the clinical data collection and retrieved patients signed informed-consent forms. EA, AH and JC contributed in the interpretation of data and critically revised the manuscript for important intellectual content. FL participated in the interpretation of data, made substantive intellectual contribution to the study and drafting the manuscript. All authors read and approved the final manuscript.

## Pre-publication history

The pre-publication history for this paper can be accessed here:

http://www.biomedcentral.com/1471-2407/14/230/prepub
